# Medicinal Plants with Anti-Leukemic Effects: A Review

**DOI:** 10.3390/molecules26092741

**Published:** 2021-05-07

**Authors:** Tahani Maher, Raha Ahmad Raus, Djabir Daddiouaissa, Farah Ahmad, Noor Suhana Adzhar, Elda Surhaida Latif, Ferid Abdulhafiz, Arifullah Mohammed

**Affiliations:** 1 Biotechnology Engineering Department, Kulliyyah of Engineering, International Islamic University, Malaysia (IIUM), P.O. Box 10, Gombak, Kuala Lumpur 50728, Malaysia; alawdat.tahani@live.iium.edu.my (T.M.); rahaar@iium.edu.my (R.A.R.); daddiouaissa.djabir@live.iium.edu.my (D.D.); farahahmad@iium.edu.my (F.A.); 2 International Institute for Halal Research and Training (INHART), Level 3, KICT Building, International Islamic University Malaysia (IIUM), Jalan Gombak, Kuala Lumpur 53100, Malaysia; 3 Faculty of Industrial Sciences and Technology, Universiti Malaysia, Pekan Pahang, Kuantan 26600, Malaysia; nsuhana@ump.edu.my; 4 Centre for Toxicology and Health Risk Studies (CORE), Faculty of Health Sciences, Universiti Kebangsaan Malaysia, Jalan Raja Muda Abdul Aziz, Kuala Lumpur 50300, Malaysia; elda.latif@ukm.edu.my; 5 Faculty of Agro-Based Industry, Universiti Malaysia Kelantan, Jeli, Kelantan 17600, Malaysia; ferid.f18e006f@siswa.umk.edu.my

**Keywords:** anti-leukemia, leukemia, medicinal plants, alternative medicine, drug discovery

## Abstract

Leukemia is a leukocyte cancer that is characterized by anarchic growth of immature immune cells in the bone marrow, blood and spleen. There are many forms of leukemia, and the best course of therapy and the chance of a patient’s survival depend on the type of leukemic disease. Different forms of drugs have been used to treat leukemia. Due to the adverse effects associated with such therapies and drug resistance, the search for safer and more effective drugs remains one of the most challenging areas of research. Thus, new therapeutic approaches are important to improving outcomes. Almost half of the drugs utilized nowadays in treating cancer are from natural products and their derivatives. Medicinal plants have proven to be an effective natural source of anti-leukemic drugs. The cytotoxicity and the mechanisms underlying the toxicity of these plants to leukemic cells and their isolated compounds were investigated. Effort has been made throughout this comprehensive review to highlight the recent developments and milestones achieved in leukemia therapies using plant-derived compounds and the crude extracts from various medicinal plants. Furthermore, the mechanisms of action of these plants are discussed.

## 1. Introduction

Leukemia is considered to be among the most common cancer types and happens because of the abnormal proliferation of white blood cells [1,2]. Leukemia can be classified as acute or chronic according to the patient’s age; and using the blood cell type into chronic (CML) or acute (AML) myeloblastic leukemia; or chronic (CLL) or acute (ALL) lymphoblastic leukemia. ALL appears to be more common in children with a high incidence between two and five years of age, whereas the other categories are more common in adults [3]. A patient suspected of having leukemia needs to consult an oncologist–hematologist to make a diagnosis and start treatment. Chemotherapy drugs, radiation and monoclonal hematopoietic stem cell transplantation are now the most common treatments for each type of leukemia. The treatment plan varies based on the subtype of leukemia, cytogenetic and molecular studies and the age of the patient. Although chemotherapeutic drugs such as cyclophosphamide, fludarabine, prednisone, chlorambucil and doxorubicin are used clinically to treat leukemia, they are used in combination instead of being used as single drugs and often do not improve the patient’s overall survival rate. Complications of intensive chemotherapeutic treatment for a patient suffering from leukemia include risk of death from cardiac and neurological complications [4], oral mucositis [5], gastrointestinal and liver toxicities [6], an infectious complication for ALL patients [7] and reduced bone mineral density at a high rate for children with ALL [8,9]. In spite of causing several side effects, the overuse of these drugs leads to chemotherapeutic drug resistance in leukemia cells [10]. Thus, the production of new therapeutic drugs having higher potency and lower toxicity is important to expanding the overall life expectancy.

Medicinal plants have a long history of being used to cure numerous illnesses and different forms of cancer [11,12,13]. Plant-based bioactive compounds are considered as renewable sources of anti-leukemic agents [14] due to their diversity and availability. Findings on the anti-proliferative effects of pure compounds and crude extracts from plants against leukemia [15] have questioned their ability to replace established drug treatments for leukemia [14]. This article reviews and discusses the current knowledge of the anti-leukemic properties of ethnomedicinal plants and discusses their potential efficacy. Additionally, the possible mechanisms of how the pure compounds and extracts exert their negative effects against leukemia are discussed as well. For this review, we developed a search strategy to identify relevant literature. The search strategy was tailored to five databases—PubMed, Medline, Web of Science, Scopus and Google scholar databases; and the search terms used were the following “Medicinal Plants with Anti-leukemic Effects” or “Cytotoxicity Effects of Medicinal Plants” or “Anti-Cancer Medicinal Plants.” All searches spanned from database inception until 2020 and included journal articles and Research Reports, published in English, full text only. The initial search strategy identified about 147 references. Of them, 113 papers were selected for further screening and met all our inclusion criteria. Each plant presented in this review is presented by explaining the some basic details about it—the taxonomy; a description; its bioactive compounds and their common biological activities, while concentrating on the anticancer and anti-leukemic effects and the mechanisms of action. ChemSkech version 12.01 Software (Advanced Chemistry Development, Toronto, ON, Canada) was used to draw the chemical structures.

## 2. The Anti-Leukemic Activity of Traditional Medicinal Plants

Many ethnomedicinal plants have been recommended to treat leukemic disease in the conventional medicine system [16]. Conventional medicine pursues the use of natural ingredients, thereby offering reinforcements to the cell regularly to revitalize the normal cell for cancer therapy [17]. This review article elaborates different species of plants which have great potential for combating leukemia. The compounds in this review include plant-derived anti-leukemic compounds clinically used as treatments, and others being examined in clinical and pre-clinical studies.

### 2.1. Medicinal Plants Clinically Used as Treatments for Leukemia

In this section, we review several traditional medicinal plants that have established clinical uses for the treatment of leukemia.

#### 2.1.1. *Cephalotaxus harringtonia*

**Figure 1:**
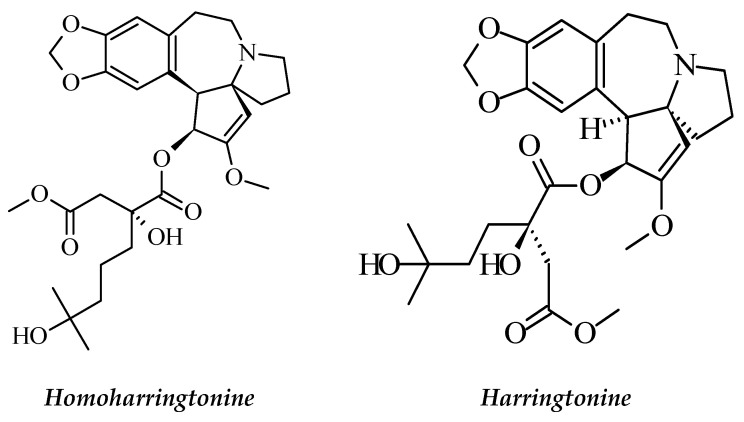
The structures of homoharringtonine and harringtonine.

*Cephalotaxus harringtonia* (family Cephalotaxaceae) is native to the southern provinces of China and is well known in traditional Chinese medicine. *C. harringtonia* is an evergreen tree consisting of ten subspecies in Asia, and the majority of them are distributed in China. Some compounds isolated from *C. harringtonia* are reported to have anti-leukemic effects. Among them, homoharringtonine and harringtonine (Figure 1) are the most popular alkaloids [18,19,20,21]. Several researchers have investigated the *Cephalotaxus*-derived alkaloids using various cancer cell lines. Takemura et al. [22] investigated the growth-inhibitory effects of harringtonine (HT) in leukemia and lymphoma cell lines, and they reported that HT was active against the HL-60 cell line. The mechanism of action involved a diversion of proliferating blast cells into a differentiation pathway, resulting in the arrest of cellular proliferation. Another study investigated the effects of homoharringtonine (HHT) on the proliferation and differentiation of human leukemic cells in vitro. Their results show that HTT inhibited colony formation of the HL-60 myeloid cell line [23]. Further studies showed that *C. harringtonia* alkaloids block peptide bond formation and aminoacyl tRNA binding, thereby inhibiting translation at elongation. Similar evidence shows *Cephalotaxus* alkaloids exert their anticancer effects by inhibiting protein synthesis at the elongation step of translation [24]. Homoharringtonine was subjected to preclinical and clinical studies and was approved by the US FDA for the treatment of patients with chronic myeloid leukemia (CML) [21,22,23,24].

#### 2.1.2. *Catharanthus roseus*

*Catharanthus roseus* (synonym of Vinca rosea). *C. roseus*, usually known as Madagascar periwinkle, belongs to the Apocynaceae family. The plant has a short stem and glossy green leaves, and flowers appear in spring and autumn (three colors: pink, purple and white) [25,26]. *C. roseus* is originally endemic to Madagascar, Jamaica and the Philippines [27].

**Figure 2:**
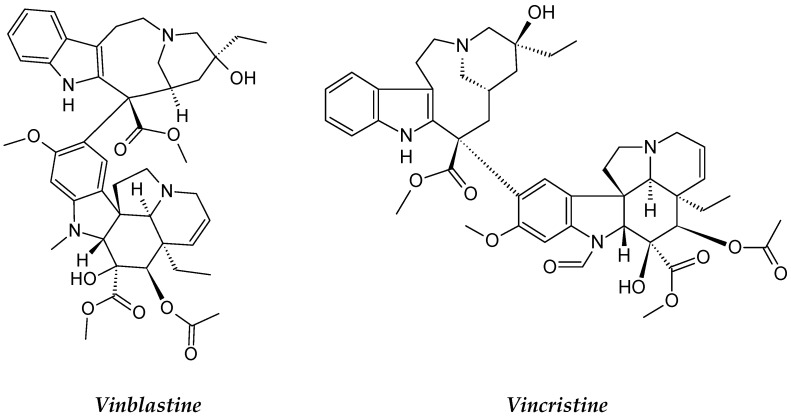
The structures of vinblastine and vincristine.

The most potentially active chemical constituents of *C. roseus* are alkaloids. More than 400 alkaloids were isolated from this plant. Vinca alkaloids vinblastine and vincristine (Figure 2) are the two major active compounds; the two semi-synthetic analogues derived from such active compounds are vinorelbine and vindesine [28,29,30]. Vincristine and vinblastine are the first plant alkaloids to be clinically used as anticancer drugs. Vincristine causes cell death in acute lymphoblastic leukemia cells without significant prior mitotic arrest [31]. Vinblastine mechanistically encourages the minus-end detachment of microtubules from their organizational centers; this generates microtubule fragments [32]. Vinblastine is regularly utilized to treat different types of cancers, including leukemia, breast cancers, small-cell lung cancer, Hodgkin’s and non-Hodgkin’s lymphoma, nephroblastoma, testicular carcinoma, germ cell tumors and Ewing’s sarcoma [30]. Vincristine is used for leukemia in children and its cytotoxicity was examined in vitro in normal peripheral blood mononuclear cells (PBMC) and CLL cells [31,32]. Vincristine is commonly used to cure a variety of carcinomas, such as multiple myeloma, B-cell lymphoma, glioma, metastatic melanoma, negative estrogen-receptor breast cancer, neuroblastoma, colorectal cancer, Hodgkin’s rhabdomyosarcoma, non-Hodgkin’s lymphoma and Wilms’ tumor [32]. The first plant-derived anticancer agents certified by the US FDA were vincristine sulphate (Oncovin^®^) and vinblastine sulphate (Velban^®^) in 1963 and 1965, respectively [28]. Vinorelbine and vindesine are used in treating a variety of cancers and can be mixed with other chemotherapeutic agents [28] to treat leukemias, lymphomas, breast and lung cancers, Hodgkin’s lymphoma, advanced testicular cancer, Kaposi’s sarcoma, bladder cancer and brain cancer [32].

### 2.2. Medicinal Plants in Clinical Trials

#### 2.2.1. *Maytenus serrata*

**Figure 3:**
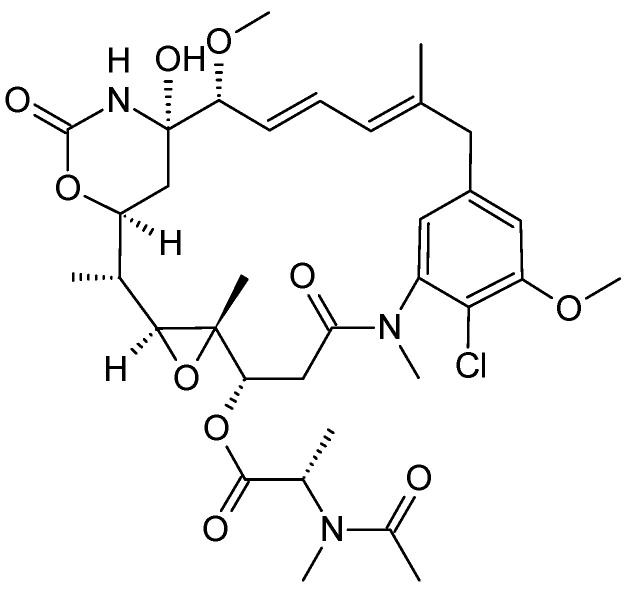
Structure of *maytansinoids.*

*Maytenus serrata*, commonly called Ethiopian shrub, is a flowering plant in the family of *Celastraceae* and is widely distributed throughout Africa, Australasia, India and South America. The bark of *Maytenus* has been used for its medicinal properties [33,34]. *Maytenus Serrata* contains many different cytotoxic compounds—for example, Maytansinoids (Figure 3), which showed inhibitory activity against P-388 lymphocytic leukemia cells [35]. Due to the high in vitro potency of the maytansinoids, further clinical trials are underway to test the effectiveness in humans. The anticancer activities of maytansinoids have been attributed to their ability to disrupt microtubule function, and they are also considered highly potent tubulin inhibitors [36,37]. In addition to its in vitro cytotoxic potential against several cancer cells, maytansine is considered as an ansamycin antibiotic, and its maytansinoid derivative exhibits cytotoxicity against many tumor cell lines—for example, acute myeloid leukemia (AML) cells lines—and it was reported to have an antileukemic effect in phase I and II clinical trials [38]. Another study of AVE9633 (huMy9-6-DM4), which is an antibody–maytansinoid conjugate and composed of a thiol-containing maytansinoid derivative, created by ImmunoGen, involved a phase 1 clinical trial in adult patients with relapsed/refractory acute myeloid leukemia, and it showed both improved efficacy and reduced systemic toxicity of anti-leukemia therapy [39].

#### 2.2.2. *Dysoxylum binectariferum*

*Dysoxylum* genus grows naturally in forests and is locally known as Achalkaat considera. It is a small or medium-sized tree up to 5 feet in girth and 50 feet high from the family Meliaceae. It is widely distributed throughout eastern and northern Australia, New Caledonia, South East Asia, Southern, New Guinea, China, the Indian subcontinent, the Philippines, Taiwan and in the western Pacific Ocean [40]. *D. binectariferum*’s fruits contain rohitukine (C_16_H_19_O_5_N), which is a precursor for the semi-synthetic derivative flavopiridol [40,41].

**Figure 4:**
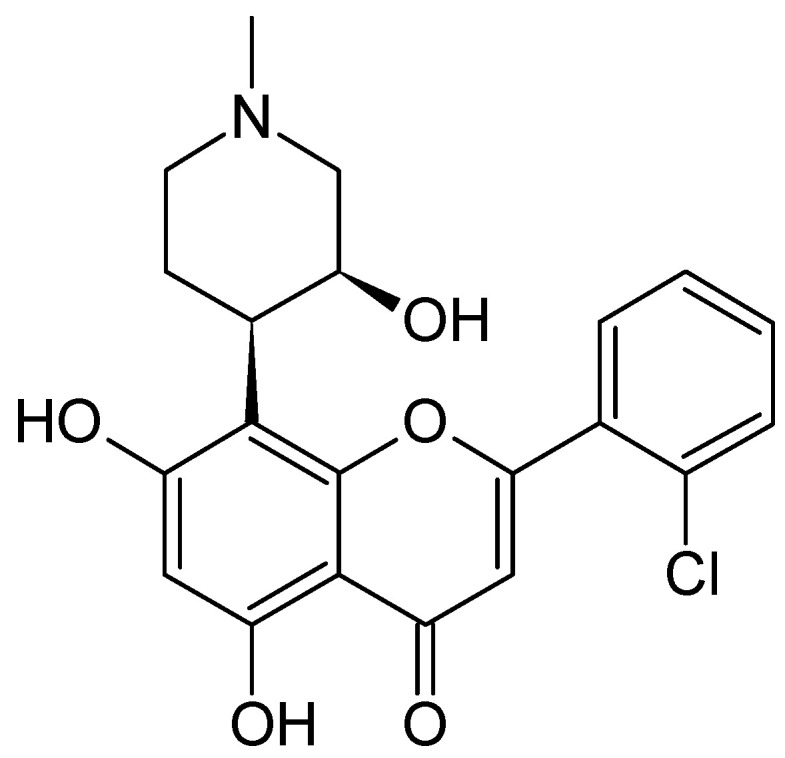
The structure of flavopiridol.

Flavopiridol is an alkaloid isolated from the stem bark of *Dysoxylum binectariferum*. Flavopiridol (Figure 4) has shown potential anti-leukemia effects [42]. Karp et al. [42] studied the anti-leukemia effects of flavopiridol. Their results show that flavopiridol is a broad spectrum cytotoxic drug against acute myeloid leukemia (AML), and its mechanism of action is through the inhibition of cyclin-dependent kinases. Flavopiridol has been used as a potent antiproliferative agent; it showed antitumor activity in clinical studies [43]. Flavopiridol induced apoptosis in bone marrow cells taken from 20 adults patients with refractory acute leukemia in vitro [44,45].

Several studies have also examined the combinational effects with a variety of anti-leukemic compounds to maximize the cytotoxic effects of flavopiridol-containing combinations [45]. For example, alvocidib (formerly flavopiridol) was evaluated for its therapeutic effect on acute myeloid leukemia (AML) alone and in combination with cytotoxic agents cytarabine and mitoxantrone (FLAM). Alvocidib showed reproducible, encouraging results in AML patients [46,47].

### 2.3. Medicinal Plants in Pre-Clinical Investigations (In Vitro) for Leukemia

In this section, we highlight various medicinal plants that are in preclinical investigations (in vitro) for the treatment of leukemia.

#### 2.3.1. *Salix* Genus (Willows)

**Figure 5:**
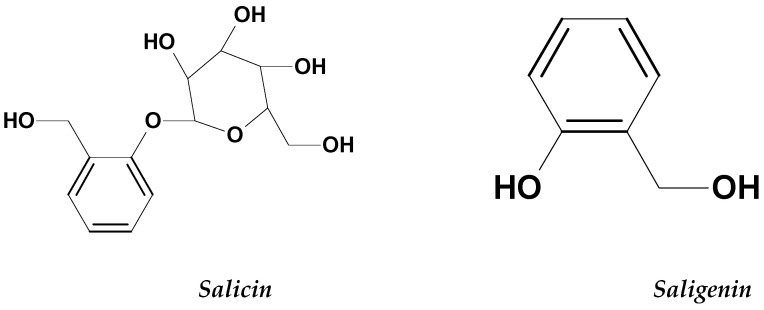
The structures of salicin and saligenin.

Willows belongs to the Salicaceae family and *Salix* genus, which contains about 400 species. It is also called osiers and sallows [48] depending on the shape of the leaves. Species with narrower lance-shaped leaves are known as osiers, and those with rounder lance-shaped leaves are known as sallows. Willows are native to cold temperate regions of the Northern Hemisphere. Their leaves and bark have been utilized in treating fevers and as a remedy for pain relief. Its pain relief effect has been studied scientifically, and the bioactive compound salicin (Figure 5) was found to be responsible for it [49,50].

Willow leaves have also been tested and evaluated for anti-leukemia activity. The aqueous extract from willow leaves has been reported to have high activity against ALL and AML cells. The extract prevented proliferation by inducing apoptosis and causing DNA fragmentation within both types of tumor cells [51]. The leaf extract is also toxic toward human leukemia 60 (HL-60) and significantly reduced the viability of the HL-60 cells in a time and dose-dependent manner [49]. The extract induced toxicity towards HL-60 cells by increasing the p53 expression in the tumor, initiating cell cycle arrest at G2M and decreasing cell division [51]. Salicin and saligenin (Figure 5) found in the leaf extract killed HL-60 cells by damaging some proteins and enzymes [49]. The anti-leukemic effects of the willow extract are also probably related to salicylic acid derivatives, phenols, flavonoids, proanthocyanidins and total tannins that are found in the extract [48].

#### 2.3.2. *Vernonia amygdalina*

*Vernonia amygdalina* belongs to the Compositae family and is commonly called an African medicinal plant [52]. The plant is original to Nigeria (West Africa) and is widely distributed in the African countries, especially in South Africa (including Eastern Cape, Mpumalanga and KwaZulu Natal) [52]. The plants usually grow in the western part of Sudan during the summer and can also be found in Asia, particularly in Singapore and Malaysia. It is the largest plant used in the genus of *Vernonia* and commonly used to treat gastrointestinal problems, wounds, hepatitis and diabetes [52]. *Vernonia* genus is the best source of sesquiterpene lactone compounds that have been stated to be cytotoxic and anticancer [53].

**Figure 6:**
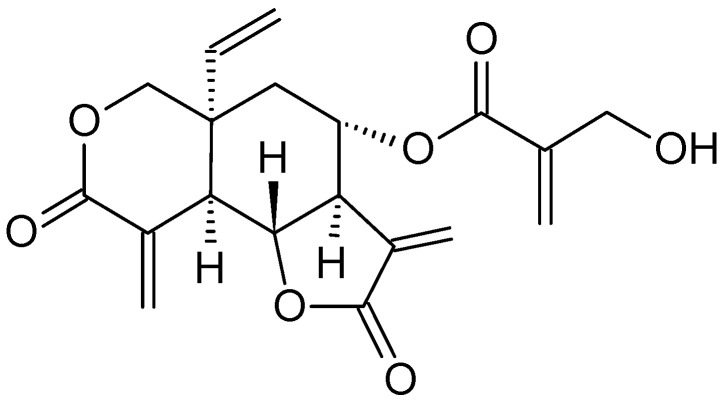
The structure of vernodalin.

In vitro testing of *V. amygdalina* root extract showed that it possessed significant antiproliferative activity and killed the majority (50–75%) of ALL and AML leukemic cells [1]. Vernodaline and vernolide (Figure 6) are sesquiterpene lactones that are commonly found in the root extract of *V. amygdalina*; they play a significant role in killing abnormal leukemic cells by promoting cell apoptosis [1,52]. A cytotoxicity study of *V. amygdalina* leaf extract showed that the extract prevents the proliferation of two cancer cell types, AML and ALL [54]. Another study observed remarkable destruction of lymphoblasts after treating mononuclear ALL cells with *V. amygdalina* extract for 24 h [1].

It has been reported that the ethanol extract of *V. amygdalina* contains phenolic and falconoid compounds, glycosides and many types of tannin. Due to this, *V. amygdalina* extract used alone or mixed with other extracts or drugs has the ability to selectively fight the leukemic cells [1]. Therefore, *V. amygdalina* extracts could constitute low-cost cancer therapy after safety and efficacy studies, and lead to drug-resistant cancer treatments [55].

#### 2.3.3. *Nyctanthes arbor-tristis*

*Nyctanthes arbor-tristis* is also known as night-flowering jasmine. It is a wild shrub-flourishing plant (Family: Oleaceae) [56], and popular medicine in India, West Bengal and Sri Lanka [56]. *N. arbor-tristis* has been tested by scientists for anti-inflammatory, antioxidant, antidepressant, antiviral, analgesic, anthelmintic, antihistaminic, amoebicidal, antipyretic and anticancer activities [57,58].

It was reported that the cytotoxic activity of seed extracts on CEM and CEM/VLB cells is through cyclin kinase protein up-regulation (WAF1/p21) and cell cycle blockage at G0/G1 phase, resulting in cancer cell death [59]. Flow cytometric analysis of *N. abor-tristis* extract-treated cells showed that the seed extracts caused sub-G0 and G0/G1 phase arrest. Recently, *N. abor-tristis* flower aqueous extract has been demonstrated to potently inhibit the cell proliferation of the human T l ymphocyte cell line [60]. The flower extract has also shown cytotoxicity against CML K562 in a dose-dependent manner and caused significant cell death in resistant and sensitive human cancer cell lines [57]. These properties induced by the plant extract are probably because of the presence of various types of phytochemicals, including phenols, tannins, steroids and glycosides. These phytochemicals are projected to be responsible for the antitumor activity [57,58]. The crude extract of *N. abor-tristis*, and pure compounds such as arbortristoside A, hydroxy hexahydrobenzofuran-one and β-hydroxyloganin that were isolated from the plant seeds, along with a polysaccharide from the leaves and naringenin from the stems, have been reported to possess antioxidant and anticancer properties [60,61]. Due to this, the water extract of *N. abor-tristis* flowers should be further investigated as a neutral medicine to stop leukemia and to be mixed with other therapeutic drugs.

#### 2.3.4. *Annona glabra*

**Figure 7:**
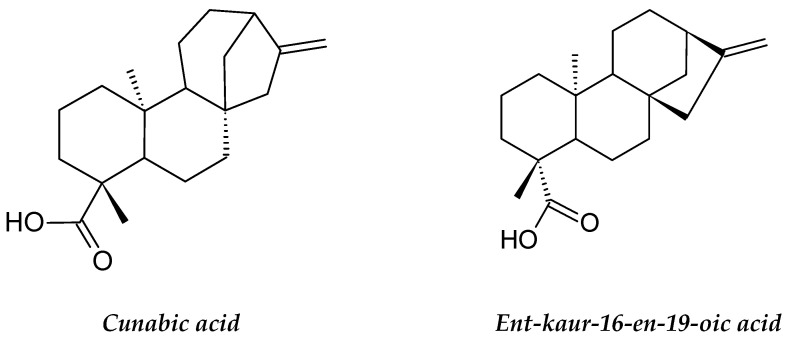
The structures of cunabic acid and Ent-kaur-16-en-19-oic acid.

*Annona glabra* L., known as the pond apple, belongs to the Annonaceae family [62]. It is a tropical wild tree native to Asia and the Americas that has been used in traditional Chinese medicine. Its phytochemical constituents, such as acetogenins; diterpenoids like cunabic acid and Ent-kaurenoic acid (Figure 7); and alkaloids, have been shown to exhibit antimalarial, anti-HIV and anticancer properties [62,63].

The anti-tumor activity of *A. glabra* was tested by using the alcoholic extracts of the leaves, pulp and seeds on leukemia cell lines [62]. The study reported that the seed extract of *A. glabra* was more effective than pulp and leaf extracts against multidrug-resistant leukemia (CEM/VLB) and drug-sensitive leukemia (CEM) cells. These findings support the traditional utilization of the alcoholic seed extract of *A. glabra* as a rich source of antitumor drugs. The methanolic extract from *A. glabra* fruits also showed a potent effect against HL-60 cells by cell cycle arrest at the G1 phase [62]. In HL-60 cells, *A. glabra* fruit extract was seen to negatively affect Bcl-2 and Bax proteins, and cleaved caspase-3 and PARP to induce cancer cell death. The fruit extract also stimulated apoptosis by reducing AKT phosphorylation and down-regulating c-myc protein [63]. Besides, Liu et al. [64] found that leaf extracts of *A. glabra* at high concentrations inhibited leukemia cell colony formation and caused more leukemia cells to become apoptotic. Leaf extracts of *A. glabra* reduced the migratory potential of leukemia cells as well. In vitro biochemical investigations on the treated-leukemia cells showed that the leaf extract also stimulated the activity of free radical scavenging and ROS and reduced ATP synthesis [64].

Screening of the phytochemical constituents of the ethanolic extracts of *A. glabra* leaves showed that the plant leaves contain terpenoids, flavonoids, saponins, glycosides, anthraquinones, steroids, tannins and acidic compounds, and do not contain alkaloids, phlobatanin or reducing sugars [64]. The methanolic extracts of *A. glabra* fruit contain dihydrophaseic acid β-d-glucopyranoside, dihydroxybenzoic acid, dimethoxyphenyl β-d-glucopyranoside, icariside D2-d-xylopyranoside, cucumegastigmane I, blumenol A and icariside B1. Among the compounds found in *A. glabra* fruit, the one responsible for the cytotoxic effect on HL-60 cell line is icariside D2 [62]. Indisputably, based on the results above, the anticancer potential of *A. glabra* extracts is strong, validating its complementary and alternative medicinal uses.

#### 2.3.5. *Basella alba*

*Basella alba* is a wildly cultivated, cool-season vegetable categorized under the family of Basellaceae [65]. *B. alba* is known as vine spinach, Chinese spinach, Indian spinach, Malabar spinach and Ceylon spinach [65]. *B. alba* is native to Malaysia, India, the Philippines, Indonesia, tropical Africa, tropical and South America, Southeast of Brazil and the Caribbean. India and China are the two major countries utilizing the plant for its medicinal benefits [66]. In India, the Ayurvedic treatment uses *B. alba* leaves against oral cancer, leukemia and melanoma [66]. The plant was reported for its anticonvulsant, anti-inflammatory and antifungal activities, and as an analgesic, an anemia treatment and an androgenic therapy [67,68].

**Figure 8:**
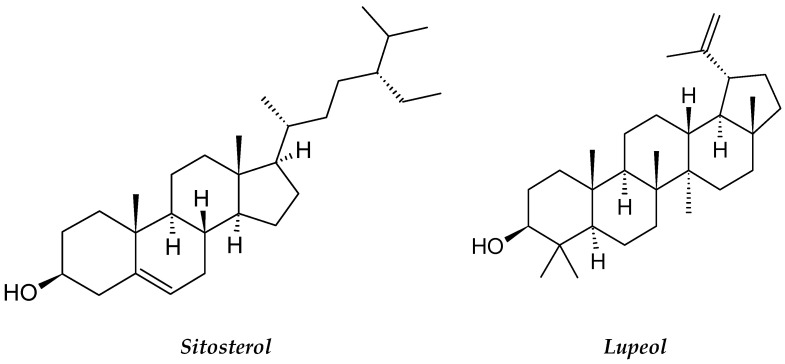
The structures of sitosterol and lupeol.

*B. alba* contains various bioactive phytoconstituents with proven cytotoxic, antioxidant, hemagglutinating and antiproliferative effects on cancer cells [69]. The *B. alba* leaf and seed extracts have different phytoconstituents, such as phenols, flavonoids, saponins, alkaloids, tannins, steroids and phytosterols [70]. The plant also has many important phytochemicals, including betacyanins, carotenoids, triterpene oligoglycosides, various organic acids, basellasapoins A–D, kaempferol and betalin [71]. A different study showed that the methanolic plant extract contained β-sitosterol and lupeol (Figure 8), which were found to be cytotoxic to lung cancer cells and leukemia cells [72]. The methanolic leaf extract of *B. alba* displayed significant growth inhibition of U937 leukemic cells in a dose-dependent manner [73].

#### 2.3.6. *Ferulago angulata*

*Ferulago angulata* also known as chovir is a Persian medicinal herb, a common plant found in Iran, Iraq, Turkey, Serbia, Greece and Macedonia [74]. *F. angulata* is from the Apiaceae family, and many *Ferulago* species have traditionally been used to treat intestinal worms, hemorrhoids, ulcers, headaches and snake bites, and act as sedatives. *F. angulata* is well studied for immunomodulatory, anti-proliferative, anti-apoptotic and cytotoxic activities against various cancer lines [75] (Table 1).

Many researchers have reported that the methanolic crude extract of *F. angulata* exhibits cytotoxicity activity, and most probably exerts the effect via an apoptosis-dependent pathway [74]. It was reported that the methanolic extract inhibited the proliferation of B-cell lymphoma (Raji) U937 cells and acute myelocytic leukemia cells (KG-1A), and suppressed them in a time and dose-dependent manner [74]. *F. angulata* also decreased cell viability and induced anti-proliferative activity against HL-60 cells [76]. Coumarin is the constituent in *F. angulata* that was identified to be the bioactive ingredient that inhibits tumor cell growth and may be considered as a herbal alternative to synthetic drugs [77]. Extensive investigations on coumarin’s anti-proliferative activity showed that it inhibits a variety of mechanisms through angiogenesis inhibition, kinase inhibition, antimitotic activity, cell cycle arrest, telomerase inhibition, heat shock protein (HSP90) inhibition, aromatase inhibition, sulfatase inhibition and monocarboxylate transporters inhibition [77].

**Table 1:** A list of traditionally used medicinal plants demonstrating cytotoxic effects in different leukemia cell lines.

Scientific Name	Family	Active Compound	Leukemia Cell Line	Mechanism of Action	References
*Annona glabra*	Annonaceae	Acetogenins Diterpenoid (Cunabic acid, Ent-kaur-en-oic acid)	Human drug-sensitive leukemia (CEM), Multidrug-resistant-derived (CEM/VLB) cell lines and HL-60 cell line	Inhibition of mitochondrial respiratory chain complex, Inhibition of proliferation and Induce apoptosis and necrosis	[62,63,64]
*Basella alba*	Basellaceae	β-Sitosterol Terpenoids (Lupeol).	U937 cell line and Jurkat cell lines	Anti-leukemic, and Growth Inhibition	[67]
*Berberis amurensis*	Berberidaceae	Alkaloids Bisbenzylisoquinoline group Berbamine,4-Chlorobenzoyl Berbamine and Berberine.	Leukemic NB4 cells, Leukemic cell line K562, Chronic myeloid Leukemia cell line KU812, Gleevec-sensitive and -resistant Ph+ Leukemia cells	Induce cell apoptosis, Apoptosis, K562-r cell growth arrest and Cell proliferation inhibition	[78,79,80]
*Blumea lacera*	Asteraceae	Alkaloids, tannins, steroids, gums. Terpenoids, diterpenoid glycoside	K562, L1210, P3HR1, Raji, U937	Antiproliferation	[81]
*Bidens Pilosa*	Asteraceae	Flavonoids	L1210, U937, K562, Raji, P3HR1,	Antiproliferation	[82,83]
*Catharanthus roseus*	Apocynaceae	Alkaloids: vincristine, vinblastine, vindesine, vinorelbine.	L1210 and P1534 Leukemia cells.	Mitotic inhibitor and Arrests the cell division which causes the death of the cells	[84]
*Coreopsis lanceolata*	Asteraceae	Flavonoids: flavanone, chalcones, and aurones 4 Methoxylanceoletin.	Leukemia HL-60 cells	Antiproliferation and Apoptosis induction	[85,86,87]
*Ferulago angulata*	Apiaceae	Phenolic Flavonoid Monoterpenes: α-pinene β-pinene	Raji, U937, AML cell lines, PBL cell line, Leukemia, lymphoma cell lines, NHL, Raji, U937, KG-1A, PBMC cell lines and HL-60 cell line.	Antiproliferation, Apoptosis induction and Autophagy and necrosis.	[88,89]
*Ficus deltoidea*	Moraceae	Flavonoid, tannins, terpenoids, phenol, proanthocyanins, lignans, alkaloids and coumarins	HL60 cell line	Apoptosis induction	[90]
*Houttuynia cordata Thunb*	Saururaceae	Flavonoids	L1210, U937, K562, P3HR1, Jurkat Leukemia cell line, Acute T lymphoblastic leukemic Molt-4 cells and Human T-cell Leukemia	Antiproliferation, Apoptosis induction through an endoplasmic reticulum stress pathway.	[91,92]
*Litchi chinensis Sonn*	Sapindaceae	Phenols Flavonoids Polysaccharides Tannins: epicatechin, proanthocyanidin B2 and proanthocyanidin B4	HL-60, U937 and K562 cell lines	Antiproliferation and Apoptosis induction	[93]
*Nyctanthes arbor-tristis*	Oleaceae	Phenols: phenol, (dimethylethyl), hydroxypyridine oxide Glycosides, tannins, phenols and steroids.	AML, CLL and Jurkat T cells, CML, K562 cell lines.	Antiproliferation andApoptosis induction	[57,58,59,60]
*Olea europaea*	Oleaceae	Phenols, oleuropeosides oleuropein/verbascoside, Hydroxytyrosol, Flavons Flavonols (rutin), catechin Phenols (vanillin, tyrosol hydroxytyrosol, caffeic acid and vanillic acid).	Jurkat, K562 cells line andHL60 cell line.	Antiproliferation, Apoptosis induction and Cell cycle arrest, apoptosis induction and differentiation	[94,95,96,97,98,99]
*Scutellaria baicalensis*	Lamiaceae	Flavonoid, Baicalein, baicalin, and wogonin.	HL-60, NB-4, THP-1, U937 cells (Blin-1, Nalm-6), lymphoma cell lines (Daudi, Raji, Ramos, NCEB1), NALM-6 cell line (human, peripheral blood, B-type human Leukemia), HL-60 cell line.	Growth inhibition induce apoptosis and cell cycle arrest, Induction of apoptosis and Dose-dependent reduction of mitochondrial metabolism.	[100,101]
*Salvia officinalis*	Lamiaceae	Flavonoids and rosmarinic acid	K-562, U937, KG-1A Cell line.	Antiproliferative, anti-migratory and antiangiogenic.	[102]
*Tithonia diversifolia*	Asteraceae	Sesquiterpene lactones (STLs) Chlorogenic acid derivatives (CAs) Flavonoids, phenolics, tannins and terpenoids	HL-60 cell line. Anti-K562, L1210, P3HR1, Raji and U937 Leukemia cells	Antiproliferation andCytotoxic.	[103,104]
*Typhonium flagelliforme*	Araceae	Hexadecanoic acid, gamma sitosterol, phytol¸ octadecadienoic acid, pentadecyne, squalene, eicosane, octacosane, and geranylgeraniol. Pheophorbide, oleic acid, campesterol, stigmasterol and sitosterol, Linoleic acid.	Murine Leukemia WEHI-cancer cell lines, P388 murine Leukemia cells, HL-60 Leukemia cells, and human T4-lymphoblastoid cell line CEMss.	Antiproliferation via apoptosis induction	[105,106,107,108,109]
*Viscum album* (Mistletoe)	Viscacea	Proteins such as lectins and polypeptides like viscotoxins	ALL, NALM-6 cell lines, Jurkat E6.1 and THP-1 cells	Apoptosis induction andG2/M cell cycle arrest.	[110]
*Vernonia amygdalina* (VA)	Compositae	Sesquiterpene lactones: vernodaline and vernolide	HL-60 cell line andALL and AML immature monocytes patients, Mononuclear cells.	Antiproliferation DNA damage, and apoptosis induction.	[1,111]
*Willows tree*	Salicaceae	Salicin and saligenin	ALL and AML cell lines b- HL-60 cell line	Apoptosis induction by causing DNA damage. Antiproliferation	[51]

#### 2.3.7. *Litchi chinensis Sonn*

*Litchi chinensis Sonn* Litchi belongs to the Sapindaceae family [112], and is known for its delicious fruit in Southeast Asia. The plant is also planted in other semitropical regions for its fruits [113]. In China, the plant is popular due to its attractive shape. Often, the fresh and dried litchi fruit extracts are made into powders to be utilized in traditional Chinese medicine and Indian herbal medicine [114].

*L. chinensis* leaf extract has been tested for cytotoxicity toward many leukemia cell lines, such as K562, U937 and HL-60, and was found to be potent against those cell lines. It was observed that the leaf extract exerted the cytotoxic effect against those cells by inducing apoptosis via activation of mitochondria-mediated caspase cascades [115]. A study on the litchi fruit pericarp extract and its constituents showed that both possess anticancer effects toward MCF7 and hepatocellular carcinoma (HCC) [115].

**Figure 9:**
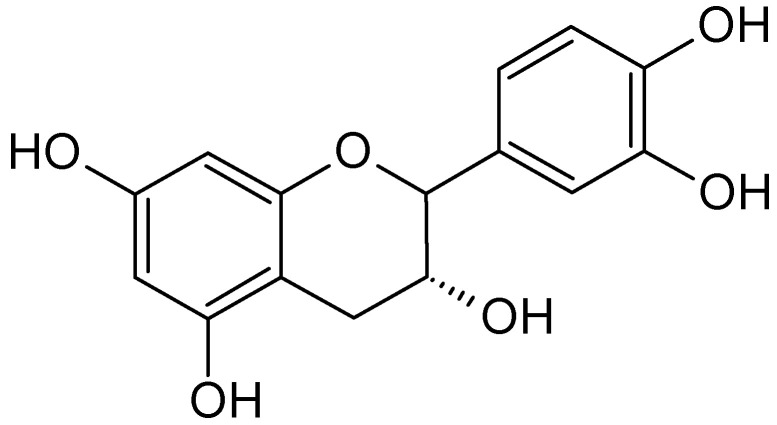
The structure of epicatechin.

A phytochemical investigation of litchis revealed a novel phenolic compound and many flavonoids, such as flavonols, isoflavones, flavones and flavanones [116,117]. Three natural flavonoids, epicatechin (Figure 9) and pro-anthocyanidin B2 and B4, have been isolated from the pericarp of *L. chinensis* and evaluated for their anticancer activities against different cancer cell lines [113].

#### 2.3.8. *Typhonium flagelliforme*

*Typhonium flagelliforme* is native to a tropical region and is in the family Araceae. It is recognized in Malaysia as the “rodent tuber”. *T. flagelliforme* is native to Bangladesh, Bhutan, Cambodia and Indonesia and is largely distributed in south India, south-east Asia and northern Australia [118]. *T. flagelliforme* juice extract is traditionally used orally to soothe coughing, swelling and more predominantly for treating different cancers by the ethnic population of Malaysia. The juice is extracted from the roots and tubers and was found to contain high doses of arginine (0.874%) and tryptophan (0.800%) [119]

**Figure 10:**
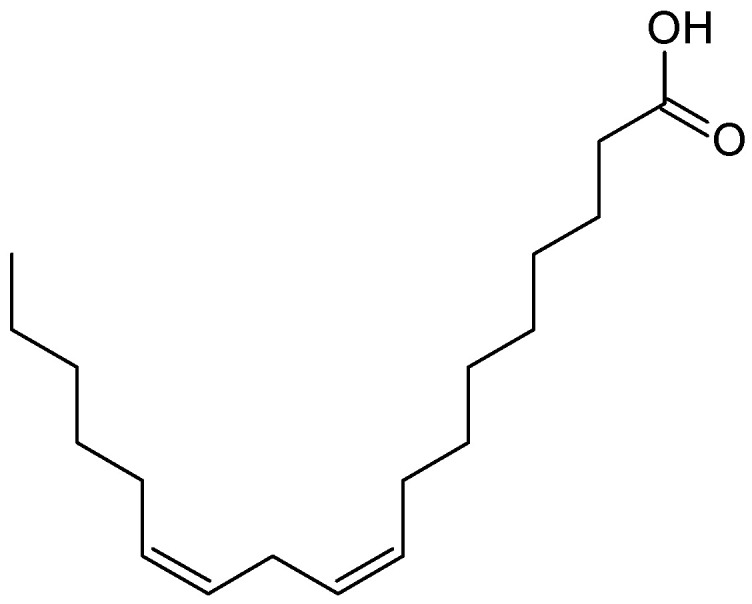
The structure of linoleic acid.

*T. flagelliforme* plant extract is considered an anticancer herbal treatment with a potential anti-leukemic effect [120]. It is also a treatment for heart trouble and has an anti-inflammatory effect. The anti-leukemic effect of *T. flagelliforme* has been tested both in vivo and in vitro in many studies. It was reported that the plant extract was effective against the murine P388 leukemia cell line [121], murine monomyelocytic and effective against WEHI-3 cells and CEM-ss cell lines [121]. Further investigation on the negative effects of *T. flagelliforme* extract, particularly of the linoleic acid-rich (Figure 10) fraction, on the leukemic CEMss cells demonstrated that the plant fraction arrested the CEM-ss cell cycle at the phase G0/G1 and induced programmed cell death by increasing caspase-3 and caspase-9 and elevating the cytochrome c in the cytosol, which lead to cleavage of poly(ADP-ribose) polymerase (PARP) [122].

The in vivo effect of *T. flagelliforme* tuber extracts demonstrated that the numbers of immature monocytes and granulocytes decreased significantly in the peripheral blood of BALB/c leukemia mice after the orally administered extract with three concentrations from 200 to 800 mg/kg for 28 days. Different chemical compounds have been isolated from *T. flagelliforme*, including methyl-13-phenyltridecanoate, phenyltridecanoic acid, coniferin, β-sitosterol, some aliphatic esters, methyl derivatives, β-daucosterol and 1-O-b-glucopyranosy l2 (hydroxyl octadecanoyl) amido-octadecadiene-diol [107]. New anticancer compounds that have shown cytotoxic activity toward breast adenocarcinoma MCF-7 cells, present in leaves and tubers, were identified by GC-MS analysis as octadecadienoic acid, gamma-sitosterol, hexadecanoic acid methyl ester, phytol¸ geranylgeraniol, eicosane, 7-pentadecyne, squalene and octacosane [107]. Another GC-MS study showed hexadecene, hexadecanoic acid and phytol and its derivative in the dichloromethane extract (D/F21) of *T. flagelliforme*, which was responsible for the inhibition of non-small cell lung carcinoma (NSCLC) cell (NCI-H23) growth. However, none of the purified compounds found above was tested against leukemia cells [122].

#### 2.3.9. *Blumea lacera*

*Blumea lacera* (Asteraceae) is commonly used as a medicinal plant in Bangladesh, and its Bengali name is Kukurshinga [123]. *B. lacera* is a yearly herb with a strong odor. Its essential oil contains cineol, fenchone and camphor [124]. *B. lacera* is largely grown in the Philippines and is traditionally used in Taiwan as a medicinal plant due to its many biological activities, including diuretic, antiscorbutic, astringent and broad anti-leukemic activities [123,124].

The *B. lacera* aqueous crude extract has been tested against various leukemic cell lines, such as K562, L1210, P3HR1, Raji and U937 [15]. The aqueous extract exhibited extensive cytotoxicity toward those cell lines, with the maximum effect on L1210 cells. Steroidal glycoalkaloid is a well-known, effective bioactive compound isolated from the methanol leaf extract of *B. lacera*. The steroidal glycoalkaloid of *B. lacera* is reported to be responsible for anti-proliferative activity against different cancers, such as human gastric carcinoma, colon carcinoma, HT-29, breast adenocarcinoma, MDA-MB-231 and MCF-7 [81]. However, the *B. lacera* steroidal glycoalkaloid has not been selectively tested against leukemia cells yet.

#### 2.3.10. *Coreopsis lanceolata*

**Figure 11:**
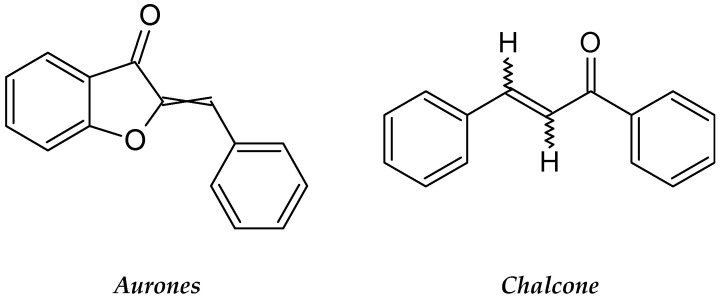
The structures of aurones and chalcone.

*Coreopsis lanceolata* is categorized under the Asteraceae family; it originates from America, South Africa and Eastern Asia. *C. lanceolata* flowers are reported to have strong bioactive compounds that effect nematodes and human leukemia cells [85]. *C. lanceolata* flowers are also an excellent natural source of rare flavonoids, including flavanone, chalcones and aurones (Figure 11), and they show strong antileukemic properties [86]. A study on the antileukemic potential of the ethyl acetate fraction of the *C. lanceolata* flowers toward HL-60 cells by using a CCK-8 assay showed that at 50 µg/mL, the extract causes 50.8% cytotoxicity towards the cells [86]. The fraction also induced apoptosis: condensation of the chromatin and fragmentation of the nuclei were observed in the cells. From the fraction, a flavonoid compound called 4-methoxylanceoletin was isolated. The compound is responsible for the anti-proliferative effect of the plant [87].

#### 2.3.11. *Tithonia diversifolia*

*Tithonia diversifolia* belongs to the Asteraceae family and is well-known as the Mexican sunflower. It is largely distributed in tropical and sub-tropical areas, such as South and North Asia, Africa, America and parts of Australia [125]. *T. diversifolia* is also considered one of the most famous Taiwanese medicinal plants and grows in the sunny grasslands of Taiwan.

Traditionally, *T. diversifolia* extracts have been used to treat diarrhea, fever, hepatitis, hepatomas, wounds and malaria [103]. Later, many researchers investigated its potential against cancer, and the first report about its anti-leukemic property was in 2002 [103]; the next was in 2004 [15]. In the first report, *T. diversifolia* extract was found to induce cellular differentiation in HL-60 cells [103]. In the second, a hot water extract of *T. diversifolia* demonstrated feeble anti-leukaemic activity against five different leukemia cell lines—U937, P3HR1, L1210, Raji and K562; and exhibited a selective cytotoxic effect [15]. Whether the existence of phytoconstituents such as phenolics, flavonoids, terpenoids and tannins in the ethanolic leaf extracts of *T. diversifolia* is responsible for its cytotoxic property [104] is not known, but many findings indicated the potential of flavonoids as novel anti-leukemia agents.

#### 2.3.12. *Bidens pilosa*

*Bidens pilosa* is among the most common plants cultivated in the tropics and subtropics around the world, having a soft, hair-like appearance, and it belongs to the Asteraceae family. It is a perennial herb that originates from South America. Folk medicinal use of *B. pilosa* has been reported in Asia, Africa, America and Oceania [126]. A phytochemical investigation indicated the existence of phenolics, phenylpropanoids, polyynes, fatty acids and flavonoid compounds in the plant. It was confirmed that these chemicals can successfully treat inflammation and tumors, and boosts the immune system [127].

Studies have shown that *B. pilosa* possesses anticancer properties, and various isolated bioactive compounds from the plant have anticancer properties. Thus, studies determining whether the plant has an anti-leukemia effect were carried out. It was found that the whole-plant water extract inhibited various leukemic cell lines, such as Raji, K562, U937, L1210 and P3HR1, in a concentration-dependent manner [15,82]. As *B. pilosa* is readily available, Yi et al. [83] suggested that the plant has the potential to be a good health supplement and a resource of natural antileukemic agents.

#### 2.3.13. *Olea europaea*

*Olea europaea*, also known as the olive, is one of the plants that is used in common, traditional Mediterranean herbal teas to treat many illnesses. *O. europaea* is in the Oleaceae family, native to coastal areas of southern Asia and Europe, and especially prevalent in the Mediterranean region [128]. *O. europaea* leaves have many effective pharmacological abilities, including neuroprotective, anti-inflammatory, antiviral, hypoglycemic, antimicrobial and anticancer effects [128].

**Figure 12:**
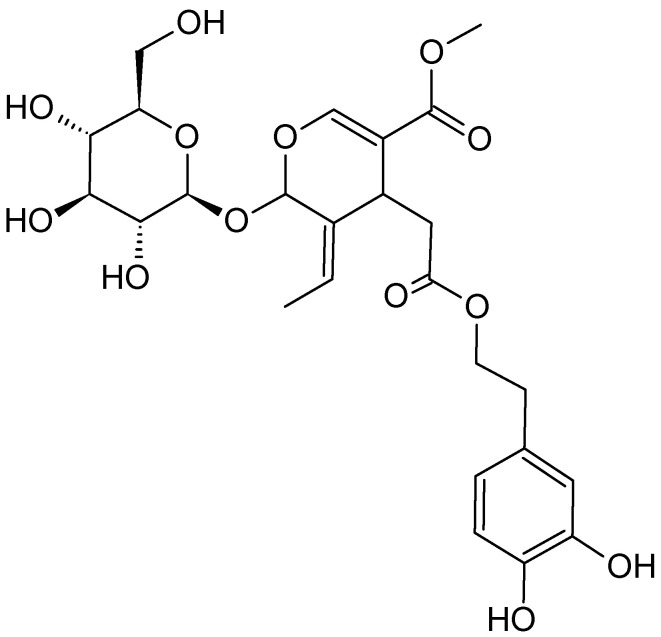
The structure of oleuropein.

An investigation on the cytotoxicity of *O. europaea* ethanol extract against Jurkat leukemic cells indicated that the extract inhibits the growth of Jurkat cells and causes apoptosis at non-cytotoxic concentrations [95]. *O. europaea* leaf extract also inhibited human chronic myelogenous leukemia K562 cell growth and caused cell cycle arrest at G0/G1 and metaphase [95]. The same leaf extract also stimulated apoptosis and monocyte/macrophage differentiation of leukemia K562 cells. A phytochemical study demonstrated the existence of several bioactive compounds, including phenols, flavonoids and secoiridoid glycosides in different parts of *O. europaea*. Oleuropein is responsible for the health benefits of *O. europaea* leaves (Figure 12), which is the major constituent of the leaves. Other phytochemical presents are flavonoids and hydroxytyrosols, such as verbascoside and luteolin-7-*O*-glucoside, which have been determined to inhibit the proliferation of cancer cells [97].

#### 2.3.14. *Houttuynia cordata* Thunb

*Houttuynia cordata* is considered one of the aromatic medicinal plants in the Saururaceae family. *H. cordata* is a local plant found in Northern Thailand, Japan, Korea, Southern China and Southeast Asia generally [129]. This plant has commonly used the world over for many medical purposes. A scientific investigation of *H. cordata* showed its extract has anti-leukemic activity against various leukemia cell lines, including K562, L1210, P3HR1 and U937 [92]. *H. cordata* extract is also cytotoxic against the human lymphoblastic leukemia cell line Molt-4, and exerts its effect in a concentration-dependent manner. The plant extract was observed to provoke apoptosis through the endoplasmic reticulum stress pathway in the Molt-4 cells [91]. The negative effect of *H. cordata* extract towards Jurkat and U937 human leukemic cells was also due to apoptosis [129].

Phytochemical screening of *H. cordata* extracts demonstrated that the plant’s extracts have many bioactive compounds, such as flavonoids, saponin, alkaloid, tannin and steroid compounds [130]. *H. cordata* flavonoids have biological activities that include antiproliferation, suppression of carcinogens, disruption of the cell cycle, activation of apoptosis, angiogenic inhibition and antioxidation, which can result in the anti-leukemic property [91,131]. *H. cordata* also contains phenolic acids called vanillic, gallic, p-hydroxybenzoic, p-coumaric, syringic, sinapinic and ferulic acids, among which, p-coumaric acid is considered as the main compound, followed by ferulic acid. These phenolic acid compounds are responsible for the cytotoxicity toward cancer cells [92]. Due to *H. cordata*’s cytotoxicity against several leukemic cell lines, researchers have suggested its utilization as an alternative medicine in the treatment of leukemia.

#### 2.3.15. *Viscum album*

*Viscum album* (mistletoe) belongs to the Viscaceae family, is distributed in Europe, southwest and central Asia and northwest Africa and has been used to treat cancer since the 1920s [132]. *V. album* contains several active phytochemicals, including viscotoxins and lectins, which are essentials in treating cancer because of their antiproliferative and apoptotic effects [133]. It also contains a class of compounds that includes phenylpropanoids, flavonoids and phenolic acids which showed anti-inflammatory and antioxidant effects. Other compounds in *V. album* include triterpenes, which have been shown to possess cytotoxic and apoptotic properties [132].

*V. album* is utilized in complementary medicine to treat cancer [134], as *V. album* extracts enhance the quality of life, improve survival, stimulate the immune system and diminish the side effects of radio and chemotherapy in cancer patients [135]. In vivo and in vitro examination of the *V. album* treatment efficacy has been conducted on ALL leukemia [110], and it was found that *V. album* aqueous extract inhibited the ALL NALM-6 cell growth. In vitro inhibition against NALM-6 cell proliferation was concentration-dependent and induced dose-dependent apoptosis that led to cell death [110,133]. Treatment with *V. album* extract at different concentrations for 4 days (by intraperitoneal injection) for severe combined immunodeficiency (SCID) mice with transplanted NALM-6 cells caused an increase in white blood cell count, indicating that mistletoe extracts have interesting therapeutic effects on hematological malignancies.

#### 2.3.16. *Salvia officinalis*

*Salvia officinalis*, also known as sage, belongs to the Lamiaceae family and is commonly distributed in the Mediterranean region [136]. It has a therapeutic history and culinary use because of its pharmacological properties. In traditional medicine, *S. officinalis* showed various pharmacological functions, namely, anti-microbial, antioxidant, hemostatic, anti-inflammatory, analgesic and antitumor activities [137].

*S. officinalis* has been used on numerous cancer cells and in numerous animal models of cancer. It has shown significant inhibition of proliferation for different cancer cells, such as CML, prostate carcinomas (PC3), MCF7, SCLC, HT29 and hepatocellular carcinoma cells [137,138]. *S. officinalis* extract also showed anti-migratory and anti-angiogenic effects against human CML cell lines, including HUVEC and K562; and B lymphoma cells, WEHI-231 [139]. Another study showed *S. officinalis* affected the proliferation of human AML (KG-1A) and U937 cells in a concentration and time-dependent manner, but no significant effect against PBMCs. An in vivo study showed the *S. officinalis* water extract possesses pro-apoptotic and anti-proliferative effects on cancer cells [136]. *S. officinalis* extract contains flavonoids and rosmarinic acid that have shown anticancer effects toward different human cancer cell lines [140]. The *S. officinalis* essential oil contains trans-caryophyllene and α-humulene, which inhibit tumor cell growth [137].

#### 2.3.17. *Ficus deltoidea*

**Figure 13:**
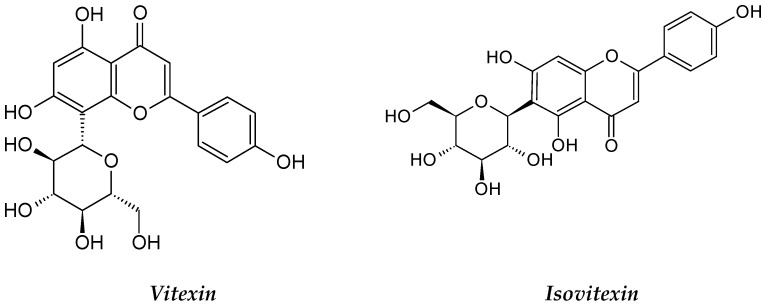
The structures of vitexin and isovitexin.

*Ficus deltoidea* belongs to the Moraceae family and was widely used as a medicinal plant. It is native to Southeast Asia [141] and distributed over many regions, such as Malaysia, Thailand, Indonesia and the Philippines [142]. In Malaysian traditional medicine, the fruit of this plant was used for headache relief, and the leaves are chewed for toothaches [143]. Often the roots and leaves of *F. deltoidea* are dried and made into a powder to be used externally for sores and wound healing [144,145]. The leaf of *F. deltoidea* contains a variety of bioactive compounds, including flavonoids, tannins, terpenoids, proanthocyanins, phenol, isoflavonoids, lignans, alkaloids and coumarins, along with vitexin and isovitexin (Figure 13) [143]. Vitexin was tested for anti-proliferative activity, and the findings revealed that vitexin causes apoptosis in different leukemia cells, such as Jurka, ALL, HL-60, AMLK-562 and CML, so this phyto-compound is likely to have a role in *F. deltoidea*’s cytotoxicity against leukemia cell lines [2]. Therefore, the high amounts of flavonoid and phenolic compounds in the leaves are responsible for the cytotoxicity of these crude extracts [146].

Moreover, *F. deltoidea* aqueous and ethanolic extracts showed cytotoxicity against different cancer cell lines, including ovarian carcinoma A2780 [146], prostate cancer PC3 [147], colon cancer (HCT 116) and breast adenocarcinoma (MDA-MB 231, MCF-7 and HCC 1937) cell lines [148]. *F. deltoidea* is also cytotoxic against human leukemia cell line HL60 and exerts this effect by causing apoptosis [89].

#### 2.3.18. *Berberis amurensis Rupr*

*Berberis amurensis Rupr* is a deciduous shrub, and a medicinal herb that belongs to the Berberidaceae plant family. It is commonly cultivated in northern Korea and the middle part of the Korean peninsula; Northeast and North China; and Russia [149]. *B. amurensis*’ fruit, leaf, root and stem extracts have been utilized in folk medicine to treat hypertonia; dysentery; eczema; inflammatory disorders; infections in the eyes; diseases of the liver, intestine and skin; and digestive and respiratory diseases. They act as hemostatic agents and fever reducers, and are utilized in the treatment of tumors [150].

**Figure 14:**
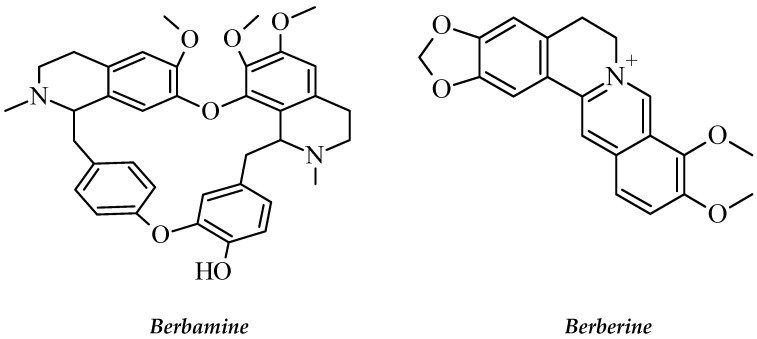
The structures of berbamine and berberine.

Alkaloids such as berberine, berbamine (Figure 14) and palmatine have been extracted from *B. amurensis* [151]. Berberine is a benzylisoquinoline alkaloid that is commonly used for the synthesis of several bioactive derivatives. A study reported that *B. amurensis* has berbamine derivatives which are considered as a new group of metabolites with anti-leukemic effects [152]. Berbamine could selectively induce programmed cell death via bcr/abl-expression in the imatinib-resistant K562 cell line and chronic myelogenous leukemia patients [149]. According to these results, *B. amurensis* could be useful in developing novel compounds to fight against leukemia.

*B. amurensis* extract can induce selectively the apoptosis of resistant and sensitive Gleevec Ph ± CML cells. It was found that berbamine extracted from *B. amurensis* inhibited the growth of tumors in CML patients [149]. It displayed selective antiproliferative activity in CML patients by inhibiting bcr/abl-positive leukemic cell growth. It was suggested that berbamine induces apoptosis of bcr/abl-positive cells (K562 cells) via the caspase-3-dependent pathway and causes p210 bcr/abl oncoprotein expression down-regulation [153]. It has been shown that other bioactive compounds in *B. amurensis* that contribute to the anti-leukemic activity are protoberberine alkaloids which belong to benzyl tetrahydro iso quinolines alkaloids [149]. Protoberine alkaloids are widely distributed in vegetables and are also utilized in traditional medicine and food supplements due to their many pharmacological properties, including antidiabetic [151], neuroprotective [153], antidepressive [154] and memory-enhancing [152] effects.

#### 2.3.19. *Scutellaria baicalensis*

*Scutellaria baicalensis* is a herbaceous species of plant belonging to the Lamiaceae family with a self-supporting growth habit. It has simple, broad leaves and is largely used in Chinese herbal medicine. It was historically used in clinical applications as an anticancer and anti-inflammatory drug. The root of the plant shows potent antitumor effects and was proposed for clinical trials with several myelomas [155].

The root of *S. baicalensis* also affects B cell leukemia and the peripheral blood leukocytes (PBLs) found in ALL patients. After treatment with *S. baicalensis* extract supplemented with baicalin, the viability of PBLs gained from ALL patients was reduced, and the NALM-6 (B-type human leukemia) cell line become apoptotic [155]. It was shown that SBE imposed a negative effect by increasing the IFNγ production in PBLs and reduced the IL-10 and TNFα production in bone marrow cells (BMC) of ALL patients, and controlled the production of cytokines, which stimulate nonspecific resistance in ALL patients and have an essential role in the innate immune system [155].

**Figure 15:**
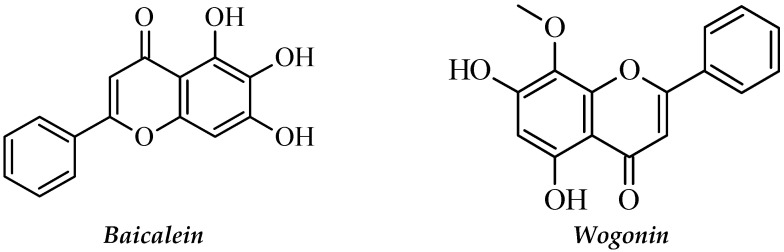
The structures of baicalein and wogonin.

Phytochemical analyses have shown that flavone compounds are major constituents in *S. baicalensis*, which include wogonin, norwogonin, baicalein and baicalin [156]. A study on wogonoside reported that it is cytotoxic toward HL-60 and U937 cell lines. Wogonoside exerts its effect through cell cycle arrest at the G1 phase and promoting differentiation. It increases the phospholipid scramblase 1 (PLSCR1) transcription, upregulates differentiation-related gene p21waf1/cip1 and downregulates oncogenic protein c-myc. Wogonin and baicalein (Figure 15), which are the aglycones of wogonoside and baicalin, respectively, also possess anticancer properties.

## 3. Conclusions

With the growing number of newly diagnosed cases of all forms of cancer globally, and in particular, hematological malignancies, there is still a strong need for novel agents that may treat or eliminate cancer. For many reasons, medicinal plants have been used to cure diverse illnesses for centuries, due to the enormous chemical diversity and biological selectivity of the bioactive compounds. This review describes the traditional uses of 20 different plants from different regions in the world that may be/have been used for the treatment of leukemia. It can be concluded that these plants’ extracts and their bioactive compounds effectively kill leukemia cells and have had similar effects in animal studies. These plant extracts and active compounds exert different mechanisms of action: the most common are suppressing proliferation; causing the arrest of the cell cycle; apoptosis; and dose and time-dependent damage to DNA. These plants may be the ideal choice for the development of adequate risk-reward studies for the potential treatment of leukemia based on clinical studies that investigate the side effects, due to easier accessibility for some populations, and better suitability as compared to chemotherapeutic drugs. Hence, more research is needed to elucidate these medicinal plants’ extracts and their active compounds’ potential for chemo-preventive and chemotherapeutic treatments by using cell line and animal studies, as well as clinical trials.

## Data Availability

The data presented in this study are available within the article.
